# Analysis of codon usage bias of WRKY transcription factors in *Helianthus annuus*

**DOI:** 10.1186/s12863-022-01064-8

**Published:** 2022-06-20

**Authors:** Yue Gao, Yan Lu, Yang Song, Lan Jing

**Affiliations:** College of Horticulture and Plant Protection, Inner Mongolia Agricultural University, Hohhot, 010011 China

**Keywords:** *Helianthus annuus*, WRKY transcription factors, Synonymous codon usage bias, Evolutionary forces

## Abstract

**Background:**

The phenomenon of codon usage bias is known to exist in many genomes and is mainly determined by mutation and selection. Codon usage bias analysis is a suitable strategy for identifying the principal evolutionary driving forces in different organisms. Sunflower (*Helianthus annuus* L.) is an annual crop that is cultivated worldwide as ornamentals, food plants and for their valuable oil. The WRKY family genes in plants play a central role in diverse regulation and multiple stress responses. Evolutionary analysis of WRKY family genes of *H. annuus* can provide rich genetic information for developing hybridization resources of the genus *Helianthus*.

**Results:**

Bases composition analysis showed the average GC content of WRKY genes of *H. annuus* was 43.42%, and the average GC3 content was 39.60%, suggesting that WRKY gene family prefers A/T(U) ending codons. There were 29 codons with relative synonymous codon usage (RSCU) greater than 1 and 22 codons ending with A and U base. The effective number of codons (ENC) and codon adaptation index (CAI) in WRKY genes ranged from 43.47–61.00 and 0.14–0.26, suggesting that the codon bias was weak and WRKY genes expression level was low. Neutrality analysis found a significant correlation between GC12 and GC3. ENC-plot showed most genes on or close to the expected curve, suggesting that mutational bias played a major role in shaping codon usage. The Parity Rule 2 plot (PR2) analysis showed that the usage of AT and GC was disproportionate. A total of three codons were identified as the optimal codons.

**Conclusion:**

Apart from natural selection effects, most of the genetic evolution in the *H. annuus* WRKY genome might be driven by mutation pressure. Our results provide a theoretical foundation for elaborating the genetic architecture and mechanisms of *H. annuus* and contributing to enrich *H. annuus* genetic resources.

**Supplementary Information:**

The online version contains supplementary material available at 10.1186/s12863-022-01064-8.

## Background

Codons consist of an arbitrary triplet of four nitrogen-containing bases. The genetic code is degenerate. Of the 64 possible codon sequences, 61 code for 20 types of amino acids that make up proteins, and the other three act as stop codons. Except for methionine (Met) and tryptophan (Trp) encoded by a single codon, the other 18 amino acids are encoded by two to six synonymous codons [1]. The selection of synonymous codons for arbitrary amino acids in different plant genomes is non-random, which is known as synonymous codon usage (SCU) bias [2]. Mutational and selective forces are considered the two main factors that affect SCU bias in different organisms [3, 4]. Nucleotide composition (G + C content) is to a large extent determined by mutational pressure and this is generally reflected in the codon usage. Highly expressed genes tend to use favored codons and exhibit high levels of codon bias [5–7]. Codon usage in highly expressed genes also has a preference for abundant tRNA species [8]. Notably gene expressivity is a major determinant of codon usage [9]. These patterns refer to natural selection for increased efficiency and accuracy of translation [8, 10, 11]. At the mechanistic level, the use of codons is shaped by the balance between mutation bias and natural selection [10, 12]. Molecular evolutionary investigations suggest that codon usage bias exists in a wide range of species from prokaryotes to eukaryotes, and may contribute to genome evolution profoundly [13]. Codon usage bias is of great importance in minimizing the chemical distances between amino acids, as the occurrence of the errors also relies on the frequency of different codons [14].

A large number of studies have shown that SCU bias is related to a variety of biological factors, including genome size [15], gene length [16], gene expression level [17], gene translation initiation signal [18], amino acid composition [19], local protein structure [20], codon context, biased gene conversion [21], recombination rate [22], tRNA abundance [23], mutation frequency and patterns [24, 25], and GC compositions [26, 27]. The coding sequences of a genome are the blueprints of gene products that provide valuable evolutionary and functional information of the organism. Thus, genome-wide investigations of codon bias patterns, and identifying the driving forces that shape their evolution are significant in genome biology studies.

Sunflower (*Helianthus annuus*) is one of the most important oil crops widely cultivated in the world. In evolutionary biology, the genus *Helianthus* is a long-term model of hybrid speciation and adaptive introgression [28]. In plant science, sunflower is a model for understanding solar tracking [29] and inflorescence development [30]. The sunflower genome (http://www.sunflowergenome.org; Genome Project Number: PRJNA64989) has now been released, and the availability of this reference genome will accelerate breeding programs as well as ecological and evolutionary research.

The WRKY family is one of the largest transcription factor families and widely involve in biotic and abiotic stress response, growth, and development of plants [31]. Lu et al. demonstrates that the codon bias of WRKY gene family in tomato (*Solanum lycopersicum*) is weak, and the codon usage bias patterns are influenced by mutation and natural selection pressure [32]. Analysis on codon usage bias of *Medicago truncatula* WRKY genes (*MtWRKY*) indicates that mutational bias is the major influence on codon usage [33]. Srivastava et al. [34] investigated the codon usage pattern of the WRKY transcription factor of the two important plant species *Arabidopsis thaliana* and *Brassica rapa*. They conclude that natural selection is the major factor guiding the evolution of different WRKY genes in both plant species.

Systematical analysis on codon usage bias of *H. annuus* WRKY gene (*HaWRKY*) has not been reported. In this study, we analyzed the codon bias and related indices of WRKY gene in sunflower and explored the factors that affected the use of synonymous codons. The knowledge is useful for understanding the evolution of codon bias and its biological significance, and provides theoretical advice to optimize the codons of WRKY genes for transgenic studies.

## Results

### Codon base composition

Multiple codon usage indices were calculated and the detailed information of the 115 WRKY gene sequences is shown in Table S1. T3s, C3s, A3s, G3s, and GC3s represent the content of T, C, A, G and the G + C at the third position of synonymous codons. T (41.24%) was the most abundant base, while A (37.94%), G (24.81%) and C (24.20%) were the second, third and fourth most abundant base according to the third base composition analysis. The average G + C content in three codon positions (GC1, GC2, and GC3) was 47.55%, 43.11%, and 39.60%, respectively. Analysis results showed that there were significant differences in G + C content in these codon sites (Table 1 shows significant differences). GC3 was lower than GC1 and GC2, and GC1 was the highest among the three codon sites. The average GC3 content was 39.60% (ranged from 29.05% to 52.58%), which was lower than the total average G + C content (GC, 43.42%). These results indicated that the codon of HaWRKY gene was dominated by A/T(U) base and preferred to end with A/T(U) base. The ENC values of the 115 genes were calculated to study the variation of HaWRKY codon usage bias. The ENC values ranged from 43.47 to 61.00, with an average of 52.63 exceeding 40, which implicated a relatively low codon usage bias. In addition, the CAI values of HaWRKY genes varied from 0.141 to 0.256, with an average value of 0.210, far less than 1, elucidating that both the codon usage bias and expression of HaWRKY genes were relatively low.

**Table 1:** Correlation coefficients of the indices influencing codon bias in HaWRKY genome

Indices	T3s	C3s	A3s	G3s	GC3s	GC1	GC2	GC3	GC	CAI	ENC
T3s	1.000										
C3s	–0.601^**^	1.000									
A3s	0.058	–0.046	1.000								
G3s	–0.333^**^	–0.237^*^	–0.646^**^	1.000							
GC3s	–0.787^**^	0.550^**^	–0.623^**^	0.657^**^	1.000						
GC1	–0.132	–0.058	–0.267^**^	0.175	0.196^*^	1.000					
GC2	–0.569^**^	0.327^**^	–0.436^**^	0.248^**^	0.594^**^	0.316^**^	1.000				
GC3	–0.773^**^	0.569^**^	–0.611^**^	0.631^**^	0.987^**^	0.192^*^	0.570^**^	1.000			
GC	–0.667^**^	0.388^**^	–0.587^**^	0.475^**^	0.804^**^	0.633^**^	0.838^**^	0.798^**^	1.000		
CAI	0.138	0.283^**^	–0.208^*^	–0.210^*^	0.044	0.148	0.121	0.033	0.129	1.000	
ENC	–0.350^**^	0.328^**^	–0.070	0.119	0.332^**^	0.276^**^	0.207^*^	0.331^**^	0.358^**^	–0.032	1.000

*Note*: ^*^ *P* value < 0.05; ^**^ *P* value < 0.01

### Correlation analysis between codon usage bias indices

Pearson Correlation Analysis showed (Table 1) that there was a significantly positive correlation between the ENC value and C3s (r = 0.328, *P* < 0.01), GC3s (r = 0.332, *P* < 0.01), GC1(r = 0.276, *P* < 0.01), GC2 (r = 0.207, *P* < 0.05), GC3 (r = 0.331, *P* < 0.01) and GC (r = 0.358, *P* < 0.01). However, the ENC value was negatively correlated with T3s (r = -0.350, *P* < 0.01). In addition, T3s was negatively correlated with C3s (r = -0.601, *P* < 0.01), G3s (r = -0.333, *P* < 0.01) and GC3s (r = -0.787, *P* < 0.01). These results indicated that the base content of the third position of the synonymous codons directly influenced the degree of codon usage preference. It is observed that genes with stronger codon bias (lower ENC value) have lower G3s, C3s and higher T3s values. Strong codon bias is often observed specifically in highly expressed genes [35]. Therefore, ENC value can be used to determine the relative expression level of the genes. The results indicated that HaWRKY genes tended to use highly expressed codons (T/A) ending with pyrimidines.

C3s had a significantly positive correlation with CAI (r = 0.283, *P* < 0.01), while A3s (r = -0.208, *P* < 0.05) and G3s (r = -0.210, *P* < 0.05) were negatively correlated with CAI. The level of gene expression can be evaluated through CAI values [36, 37]. The results suggested that the content of the third base of the synonymous codons was closely related to gene expression such that C3s was positively correlated with gene expression, while A3s and G3s were negatively correlated with gene expression.

### RSCU values analysis and determination of putative optimal codons

The program GCUA (version 1.2) (ftp://ftp.nhm.ac.uk/pub/gcua) was used to calculate RSCU values, as shown in Table 2. The results showed that 29 codons had RSCU values greater than 1 and 31 codons had RSCU values less than 1. The preferred codons were U-ended (13), A-ended (10), G-ended (4) and C-ended (2). It is worth noting that the U-ended codons, the most preferentially used among synonymous codons, were similar with the result of the T-base described above. These results supported the evidence that HaWRKY gene codons tended to end with A/T, suggesting that synonymous codon usage patterns of HaWRKY gene were biased and were influenced by compositional constraints. At the same time, there were four under-represented codons (average RSCU value < 0.6) GAC, GGC, CCC and CGC, and only one over-represented codon (average RSCU value > 1.6) AGA in the whole genome.

**Table 2:** Codon usage and high frequency used codons in HaWRKY genome

Amino acid	Codon	Frequency	Number	RSCU	Amino acid	Codon	Frequency	Number	RSCU
Ala (A)	**GCA**	**12.49**	**515**	**1.22**	Pro (P)	**CCA**	**21.80**	**899**	**1.46**
	GCC	8.32	343	0.81		CCC	8.20	338	0.55
	GCG	7.03	290	0.69		**CCG**	**15.74**	**649**	**1.05**
	**GCU**	**13.02**	**537**	**1.27**		CCU	14.07	580	0.94
Cys (C)	UGC	8.46	349	0.92	Gln (Q)	**CAA**	**34.17**	**1409**	**1.39**
	**UGU**	**9.99**	**412**	**1.08**		CAG	14.84	612	0.61
Asp (D)	GAC	12.17	502	0.56	Arg (R)	**AGA**	**20.71**	**854**	**1.95**
	**GAU**	**30.97**	**1277**	**1.44**		**AGG**	**13.36**	**551**	**1.26**
Glu (E)	**GAA**	**27.84**	**1148**	**1.24**		CGA	8.68	358	0.82
	GAG	17.02	702	0.76		CGC	4.51	186	0.43
Phe ( F)	UUC	13.29	548	0.82		CGG	9.56	394	0.90
	**UUU**	**19.23**	**793**	**1.18**		CGU	6.84	282	0.64
Gly (G)	**GGA**	**15.86**	**653**	**1.28**	Ser (S)	AGC	12.47	514	0.78
	GGC	7.15	295	0.58		**AGU**	**16.27**	**671**	**1.02**
	GGG	10.67	440	0.86		**UCA**	**23.35**	**963**	**1.47**
	**GGU**	**15.69**	**647**	**1.27**		UCC	11.11	458	0.70
His (H)	CAC	15.30	631	0.86		UCG	12.15	501	0.76
	**CAU**	**20.13**	**830**	**1.14**		**UCU**	**20.06**	**827**	**1.26**
Ile (I)	AUA	15.76	650	0.91	Thr (T)	**ACA**	**23.86**	**984**	**1.40**
	**AUC**	**18.02**	**743**	**1.04**		**ACC**	**17.73**	**731**	**1.04**
	**AUU**	**18.21**	**751**	**1.05**		ACG	11.47	473	0.67
Lys (K)	**AAA**	**36.01**	**1485**	**1.10**		ACU	15.28	630	0.89
	AAG	29.66	1223	0.90	Val (V)	GUA	10.02	413	0.73
Leu (L)	CUA	13.24	546	0.92		GUC	8.95	369	0.65
	CUC	12.27	506	0.85		**GUG**	**16.30**	**672**	**1.18**
	CUG	12.34	509	0.85		**GUU**	**19.93**	**822**	**1.44**
	**CUU**	**17.07**	**704**	**1.18**	Trp (W)	UGG	13.56	559	1
	UUA	14.02	578	0.97	Tyr (Y)	UAC	11.47	473	0.90
	**UUG**	**17.70**	**730**	**1.23**		**UAU**	**14.11**	**582**	**1.10**
Met (M)	AUG	30.22	1245	1	Terminator	UAA	5.53	228	
Asn (N)	AAC	24.91	1027	0.97		UAG	5.09	210	
	**AAU**	**26.65**	**1099**	**1.03**		**UGA**	**8.12**	**335**	

*Note*: The highest frequency used codons (RSCU value > 1) are in bold. RSCU, the relative synonymous codon usage value

By comparing the RSCU values of HaWRKY’s two bias libraries, three optimal codons GCA (Ala), AGU (Ser) and ACU (Thr) were determined whose ΔRSCU are greater than 0.3 with RSCU > 1 in high-bias genes and < 1 in low-bias genes (Table 3).

**Table 3:** The codons statistics with high and low expression genes of HaWRKY genome

Amino acid	Codon	High expressed gene		Low expressed gene		ΔRSCU
		**Frequency**	**RSCU**	**Frequency**	**RSCU**	
Ala (A)	GCU	24	1.68	39	1.25	0.43
	GCC	11	0.77	36	1.15	-0.38
	**GCA**	**20**	**1.4**	**31**	**0.99**	**0.41**
	GCG	2	0.14	19	0.61	-0.47
Cys (C)	UGU	24	1.37	21	1.2	0.17
	UGC	11	0.63	14	0.8	-0.17
Asp (D)	GAU	92	1.63	83	1.18	0.45
	GAC	21	0.37	58	0.82	-0.45
Glu (E)	GAA	77	1.34	82	1.13	0.21
	GAG	38	0.66	63	0.87	-0.21
Phe (F)	UUU	40	1.29	43	1.19	0.1
	UUC	22	0.71	29	0.81	-0.1
Gly (G)	GGU	32	1.66	46	1.45	0.21
	GGC	7	0.36	20	0.63	-0.27
	GGA	24	1.25	37	1.17	0.08
	GGG	14	0.73	24	0.76	-0.03
His (H)	CAU	36	1.6	43	1.13	0.47
	CAC	9	0.4	33	0.87	-0.47
Ile (I)	AUU	29	1.21	33	1.09	0.12
	AUC	28	1.17	32	1.05	0.12
	AUA	15	0.63	26	0.86	-0.23
Lys (K)	AAA	72	1.04	90	1.15	-0.11
	AAG	67	0.96	66	0.85	0.11
Leu (L)	UUA	27	1.4	25	1.15	0.25
	UUG	23	1.19	30	1.38	-0.19
	CUU	31	1.6	27	1.25	0.35
	CUC	10	0.52	19	0.88	-0.36
	CUA	20	1.03	23	1.06	-0.03
	CUG	5	0.26	6	0.28	-0.02
Met (M)	AUG	46	1	53	1	0
Asn (N)	AAU	52	1.21	65	1.01	0.2
	AAC	34	0.79	64	0.99	-0.2
Pro (P)	CCU	34	1.27	44	1.06	0.21
	CCC	14	0.52	27	0.65	-0.13
	CCA	45	1.68	54	1.3	0.38
	CCG	14	0.52	41	0.99	-0.47
Gln (Q)	CAA	69	1.57	88	1.49	0.08
	CAG	19	0.43	30	0.51	-0.08
Arg (R)	AGA	52	4	26	1.33	2.67
	AGG	13	1	30	1.54	-0.54
	CGU	3	0.23	15	0.77	-0.54
	CGC	0	0	7	0.36	-0.36
	CGA	6	0.46	21	1.08	-0.62
	CGG	4	0.31	18	0.92	-0.61
Ser (S)	**AGU**	**46**	**1.48**	**35**	**0.77**	**0.71**
	AGC	18	0.58	31	0.68	-0.1
	UCU	39	1.25	68	1.49	-0.24
	UCC	20	0.64	40	0.88	-0.24
	UCA	39	1.25	68	1.49	-0.24
	UCG	25	0.8	32	0.7	0.1
Thr (T)	**ACU**	**34**	**1.37**	**43**	**0.99**	**0.38**
	ACC	26	1.05	56	1.29	-0.24
	ACA	34	1.37	44	1.02	0.35
	ACG	5	0.2	30	0.69	-0.49
Val (V)	GUU	37	1.41	44	1.43	-0.02
	GUC	14	0.53	22	0.72	-0.19
	GUA	23	0.88	17	0.55	0.33
	GUG	31	1.18	40	1.3	-0.12
Trp (W)	UGG	16	1	21	1	0
Tyr (Y)	UAU	34	1.24	39	1.07	0.17
	UAC	21	0.76	34	0.93	-0.17
Terminator	UAA	5	2.5	3	1.29	1.21
	UAG	0	0	2	0.86	-0.86
	UGA	1	0.5	2	0.86	-0.36

*Note*: Optimal codons (ΔRSCU ≥ 0.3, with RSCU > 1 in high-bias genes, RSCU < 1 in low-bias genes) are in bold

### Neutrality plot analysis

**Fig. 1:**
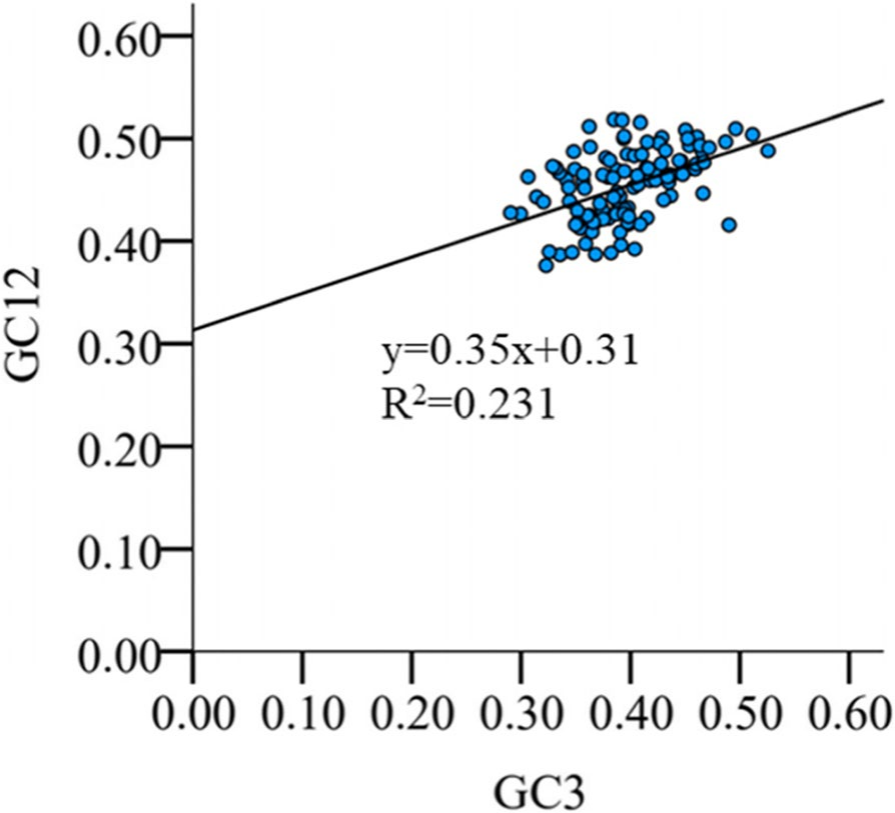
Neutrality plot analysis in HaWRKY genome. Note: GC12, the average G + C content at the first and second codon positions; GC3, the G + C content at the third codon positions

Based on the neutrality graph, the relationship between GC12 and GC3 was analyzed, and the factors of natural selection and mutation pressure in codon usage patterns were discussed. A significant correlation between GC12 and GC3 values implies that mutational pressure is superior to translation selection in the formation of codon usage bias while the non-significant correlation between them suggests that translation selection plays a dominant role in codon usage preference [38–40]. Neutral mapping analysis (Fig. 1) showed that most of the HaWRKY genes were near the standard curve and a few above or below the curve. Pearson correlation analysis showed that the correlation between GC1 and GC2 was very strong (r = 0.316, *p* < 0.01), and GC12 exhibited a significant positive correlation with GC3 (y = 0.35x + 0.31; R^2^ = 0.231; *P* < 0.01) (Fig. 1), suggesting that the effect of directional mutation pressure is present at all codon positions. Moreover, the slope of the regression line of the entire coding sequence is 0.35 which revealed that the bias of codon usage was mainly affected by mutation pressure.

### ENC and GC3s scatter plot (ENC-plot)

**Fig. 2:**
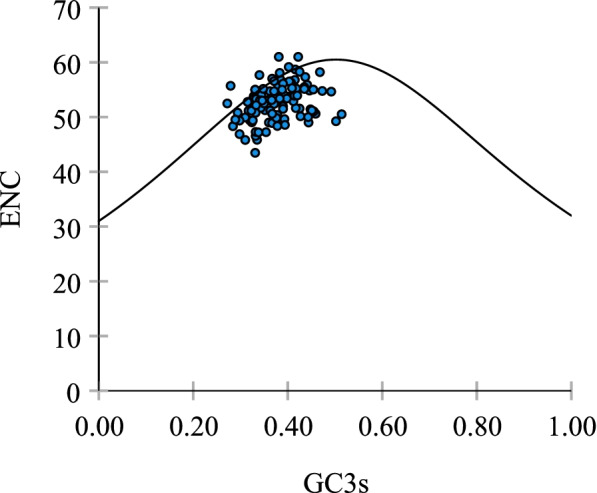
ENC-plot analysis in HaWRKY genome. The solid curve represents the expected positions of genes when the codon usage was only determined by the GC3s composition. Note: ENC, effective number of codons; GC3s, the G + C content at the third position of synonymous codons

The ENC plot was used to analyze the codon usage variation in the 115 HaWRKY CDSs (Fig. 2). Because ENC is constrained to the G + C content of the genes, investigations of codon usage patterns were performed by plotting against the GC3s of the gene [2, 41]. The solid curve represents the expected position of CDSs with codons determined only by the GC3s. When the usage of codons is limited only by G + C mutation bias, the genes represented by points in the ENC-GC3s plot should be just on the solid curved line [42]. As shown in Fig. 2, in the ENC-GC3s plot, most points were on or very close to the expected curve, suggesting that G + C mutation bias played a major role in the codon usage of the HaWRKY genes. Few points deviated well below the expected curve, suggesting that these genes should have additional codon usage biases that were independent of compositional constraints.

**Fig. 3:**
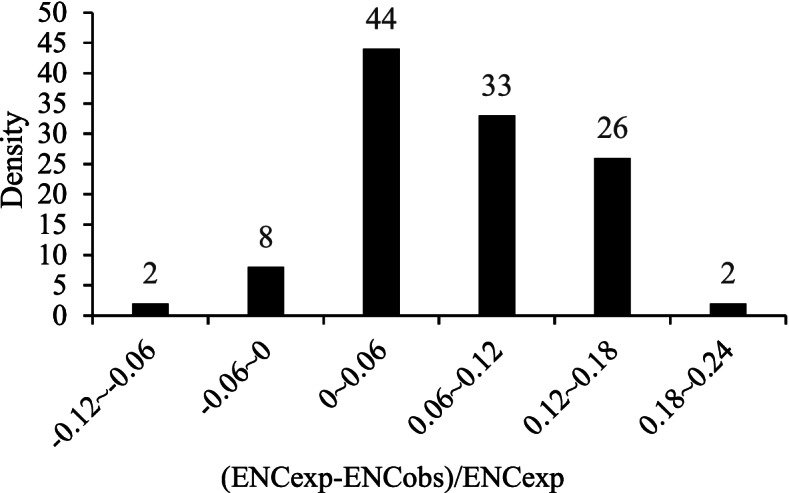
Frequency distribution of (ENCexp-ENCobs)/ENCexp, ENCexp represents expected ENC values and ENCobs represents observed ENC values

To obtain a more accurate estimation of the differences in ENC values, ENCexp-ENCobs)/ENCexp of the HaWRKY genes was calculated, and the frequency distribution was shown in Fig. 3. Of the 115 HaWRKY genes, 10 genes (8.70%) had (ENCexp-ENCobs)/ENCexp value below 0, and the other 105 genes (91.30%) had (ENCexp-ENCobs)/ENCexp value above 0. However, the (ENCexp-ENCobs)/ENCexp values for most of the HaWRKY genes (75.66%) were between –0.12 ~ 0.12, indicating that most observed ENC values were close to the expected values, which further demonstrated the *HaWRKY* codon bias was closely related to GC3s, and mainly affected by mutation pressure.

### PR2-bias plot analysis

**Fig. 4:**
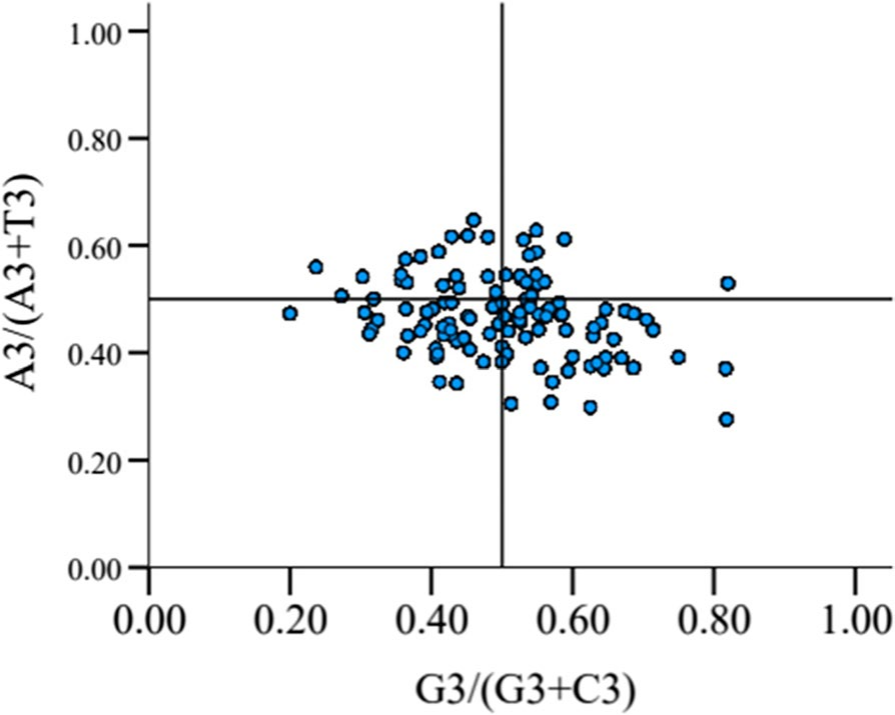
Analysis of PR2-plot in HaWRKY genome. The mean value of A3/(A3 + T3) is 0.4678, and that of G3/(G3 + C3) is 0.5033. The curves show the center line on 0.5. Note: A3/(A3 + T3), the ratio of A against A + T at the third position of codons; G3/(G3 + C3), the ratio of G against G + C at the third position of codons

Four-codon amino acids including alanine, glycine, proline, threonine, valine, arginine (CGA, CGU, CGG, CGC), leucine (CUA, CUU, CUG, CUC) and serine (UCA, UCU, UCG, UCC) were analyzed by PR2 plot (Fig. 4). It showed that most of the genes were in the lower left or lower right region along the ordinate (A < T). An almost equal number of genes were distributed on both sides (left and right) along the abscissa. The average values of A3/(A3 + T3) and G3/(G3 + C3) of eight amino acids were 0.4678 and 0.5033, respectively. The average value of A3 + T3 and G3 + C3 were 0.6329 and 0.3671, respectively. These results revealed an imbalance in the codon usage of A + T and G + C at the third base sites, suggesting that not only mutation but also selection and other factors determined the usage pattern of codons.

## Discussion

The transformation of genetic information from mRNA to protein depends on codon formation [43]. The unequal use of synonymous codons for the same amino acid can be reflected by SCU bias, which differs among various species and genes [44]. The possible causes of SCU bias have been studied in the genomes of many living organisms, for example, in *Zea mays* [45], *A. thaliana* [46], *Brachypodium distachyon* [47], *Citrus* and *Poncirus trifoliata* [48], cotton [49], *Citrus* spp. [50] and many others.

The usage pattern of the third base of the codon is closely related to codon bias [51]. The GC composition has been shown to drive codon and amino acid usage that the GC content of the third base of a codon (GC3) is considered most likely to directly reflect codon usage patterns [52]. Previous studies have shown that dicots and monocots tended to use A/U and C/G as ending codons, respectively [53]. Our study showed that the average GC content and GC3 content of HaWRKY codons were 43.42% and 39.60% respectively, indicating that the codon of HaWRKY gene of sunflower also preferred to end with A/T(U) base. This was consistent with the results of RSCU analysis of HaWRKY gene. WRKY gene families in other plants, such as *A. thaliana* [54], *Solanum lycopersicum* [32], *Ginkgo biloba* [55] and *Brassica napus* [56] preferred the codons ending with A/T(U) base as well, while WRKY gene in *Oryza sativa* preferred the codons ending with G/C [54], and *M. truncatula* with C/T(U) [33]. ENC and CAI are two parameters related to gene expression level. In this study, the ENC value of HaWRKY gene family was larger, while the CAI value was smaller, indicating that the expression level of WRKY gene family was lower in *H. annus*. This is consistent with the studies that most WRKY family genes exhibited stress-induced expression patterns [57, 58].

Codon usage bias is mainly affected by mutation pressure and natural selection [11, 59]. However, the main factors affecting codon usage bias vary greatly among different species. Neutrality plots (GC12 vs. GC3) were used to analyze the relationships between the three codon positions. In this study, there was a significantly positive correlation between the GC12 and GC3 of HaWRKY genes (r = 0.48, *P* < 0.01), indicating that GC mutational bias resulted in similar GC content at all codon locations. In addition, there were a wide range of the GC3s value of GC content in HaWRKY gene (0.272–0.514), indicating that mutation pressure was the main factor affecting codon usage.

According to the parity rule 2 analysis, the content of AT at the third position of codons was higher than that of GC. In the third position of codons of HaWRKY genes, A and T were used more frequently than G and C. This suggested that natural selection was one of the reasons for HaWRKY codon usage bias.

ENC-plot analysis showed that the ENC values of most genes were close to the expected ENC values, suggesting that the codon bias of these genes was related to GC3s, and mutation was the main influencing factor. A few points (such as HaWRKY51, HaWRKY91 and HaWRKY109) lay well below the expected curve, indicating that the codon deviations of these genes were mainly influenced by natural selection.

Based on neutral plot analysis, ENC-plot analysis and PR2 plot analysis, mutation and natural selection and other factors jointly affected the codon usage bias of HaWRKY genes, and mutation pressure played a major role, which is consistent with the previous study on WRKY in *M. truncatula* [33] and *O. sativa* [54]. Codon usage bias of genes is subject to natural selection stress and mutational stress, butmutation is especially important. Similar results have been found in micro-organisms such as baculovirus [60], herpes virus [61] and *Bacillus subtilis* [62] through whole genome analysis. Moreover, studies in *Gallus gallus* [59] and Humans [63] indicated that mutation pressure was the main driving force of codon usage bias.

Kawabe and Miyashita [64], Ingvarsson [65] and Morton and Wright [66] analyzed dicotyledons such as tobacco (*Nictiana Tabacum*), pea (*Pisum sativum*), poplar (*Populus Tremula*) and *Arabidopsis*. It was found that the codon preference of nuclear genes was mainly influenced by natural selection pressure during evolution. However, Zhang et al. reported that the codon usage bias of soybean (*Glycine max*) nuclear gene was mainly affected by mutation pressure [67]. These results suggest that codon usage preferences of nuclear genes in dicotyledon vary among plants. From the above analysis, it can be seen that different genomes can be affected by various pressures leading to codon usage preferences.

The optimization of codon usage allows the improvement of translational efficiency with modified codon usage genes in the host organism [68], and it has been introduced into many heterologous systems [69–71]. Generally speaking, genes in the GC-rich genome preferentially use codons ending with G and C, while those in the AT-rich genome prefer A and T ending codons [72]. As we found in this study, the three optimal codons (GCA, AGU and ACU) of *HaWRKY* are ended by either A or U, which is consistent with rich A + T content in HaWRKY genome. The study on MtWRKY genes identified four optimal codons, which exclusively end with G or C, while MtWRKY genome is rich in A + T content [33]. 27 optimal codons were identified in rice WRKY genes ending with G or C, and 11 optimal codons found in Arobdopis WRKY genes prefer ending with G, T or A [54]. This phenomenon is important for codon modification to enhance the expression level of foreign proteins in host cells.

## Conclusions

In this study, 115 CDSs of the *H. annus* WRKY genes were selected to analyze the SCU bias with CUSP program and codonW program, and the possible factors that influence SCU bias were inferred. With the exception of natural selection effects, the majority of genetic evolution in the *H. annuus* WRKY genome was probablydriven by mutation pressure. Our results provide a theoretical foundation for further elucidating its mechanism of evolution, degenerate primers design and study of appropriate exogenous expression systems.

## Materials and methods

### Sequence of WRKY gene family in sunflower

All of the CDS sequences and protein sequences of *H. annuus* were downloaded from the National Centre for Biotechnology (NCBI) sunflower genome database (https://www.ncbi.nlm.nih.gov/genome/?term=txid4232[orgn), GenBank assembly accession: GCA_002127325.2) in FASTA format.

WRKY transcriptional factors are defined by the presence of the conserved WRKY domain. The PFAM database (https://pfam.xfam.org/) was used to identify sequences containing WRKY domain (PF03106, http://pfam.xfam.org/family/PF03106). If there are multiple transcripts of the same gene, the longest sequence will be selected. Finally, 115 *H. annuus* WRKY genes (*HaWRKY*) were identified in total. The accession numbers and other details for the selected genomes were listed in Table S1.

### Statistical analyses

#### Codon usage bias indices

The program codonW (1.4.2 version) (http://codonw.sourceforge.net/) was used for computing effective number of codons (ENC), codon adaptation index (CAI), relative synonymous codon usage (RSCU), the total G + C contents of the entire gene (GC), the G + C content at the third position of synonymous codons (GC3s), and the content of T, C, A and G at synonymous third codon positions (T3s, C3s, A3s, G3s). By using the CUSP statistical program (https://www.bioinformatics.nl/cgi-bin/emboss/cusp), the G + C content at first, second, third codon positions represented as GC1, GC2, GC3 respectively and the average GC content at first and second codon positions (GC12) were calculated. The correlation between nucleotide contents was calculated using SPSS 23.0 statistical software. A calculation of Pearson correlation coefficient was performed. ENC value was calculated to measure the degree of deviation from equal use of synonymous codons of the ORF of the HaWRKY members. The ENC value ranges from 20 (when only one synonymous codon is selected for the corresponding amino acid) to 61 (when all synonymous codons are used identically) [73], reflecting the degree of codon usage bias. If the ENC value is greater than 40, the codon usage bias is considered low [2].

The codon adaptation index (CAI) is a geometric mean of the relative usage of codons in a gene, which is used to measure the adaptiveness of a gene towards the codon usage of highly expressed genes [74]. The values of CAI range from 0 to 1. Sequences with higher CAI values are considered to have better adaptiveness.

#### Analysis of RSCU

The RSCU value is the ratio of the actual observed value of the codon to the theoretical expected value, reflecting the relative usage preference of specific codon compositions encoding the same amino acid [75]. When RSCU = 1, codon usage is unbiased, and codon selection is equal or random. If RSCU > 1, codon usage is biased and is defined as the preferred codon. If RSCU < 1, codon usage is biased and is defined as a codon with low preference. In addition, the synonymous codons with RSCU values > 1.6 and < 0.6 are regarded as over-represented and under-represented codons respectively [76, 77]. AUG, UGG, and the three stop codons (UAG, UAA, and UGA) did not have synonymous codons and were excluded from the RSCU analysis [78].

#### Determination of putative optimal codons

The optimal codon is the preferred codon determined by calculating and sequencing the ENC values of all genes. 5% of genes with extreme low and high ENC values were regarded as two datasets (i.e. high and low expression, respectively) In order to determine the optimal codons, the RSCU values of the codons in the two databases were compared. If the difference (ΔRSCU) is equal to or greater than 0.3 and RSCU > 1 in high-bias genesand < 1 in low-bias genes, the optimal codons are defined [67]. SPSS V23.0 was used for statistical analysis.

#### Neutrality plot analysis

Dominant factors affecting codon usage bias (natural selection or mutational pressure) were analyzed by neutrality plot mapping [79] and relationships between GC12 and GC3 values of all genes were thus measured. In the neutral graph, the ordinate is the value of GC12 and the horizontal axis is the value of GC3 [80]. If the coefficient of GC3 is statistically significant and close to 1, mutation pressure is considered to be the main force affecting codon usage. The effect of mutation pressure on codon usage decreases with slope approaching 0. The slope = 0 means that the codon usage bias is completely caused by natural selection [79]. The linear relationship between GC3 variables and GC12 variables was estimated using R (version 3.6.2) [81].

#### PR2-plot analysis in HaWRKY transcription factors

Parity Rule 2 (PR2) analysis was used to estimate the effects of natural selection and mutation pressure on codon usage. The ordinate is [A3/(A3 + T3)] value, and the abscissa is [G3/(G3 + C3)]. The center of the plot is 0.5 (x = 0.5, y = 0.5), which indicates that A = T, G = C (PR2). From the degree of PR2 bias, the chain bias influenced by mutation, selection, or both can be estimated [82]. Points at the center indicate that there is no deviation between selectivity and mutation events. If genes are evenly distributed across the plane plan, i.e. if A + T and G + C have the same frequency of codon usage at the third position, then the codon usage preference is likely to be entirely caused by mutations [83]. The PR2 plots were figured by Matlab R2016a (https://www.mathworks.com/).

#### ENC-plot analysis in HaWRKY transcription factors

ENC-plot (ENC vs GC3s) was drawn by Matlab R2016a to detect the codon usage patterns between genes. The expected ENC values of GC3s were calculated as ENC = 2 + GC3s + 29/(GC3s^2^ + (1-GC3S)^2^) [2, 84]. When codon bias is affected only by mutation, genes will be distributed along or close to the standard curve, while when codon bias is affected by selection and other factors, genes will fall below the standard curve [2, 49].

If the expected ENC values is close to the observed ENC value of GC3s, it means codon bias is closely related to GC3s, and mutation is the main factor influencing codon bias. In order to better evaluate the differences in ENC values, (ENC_exp_–ENC_obs_)/ENC_exp_ of genes were calculated.

## Supplementary Information

**Additional file 1: Table S1. **The composition indices values of codon usage in HaWRKY genome 

## Data Availability

The datasets generated and analysed during the current study are available in the NCBI, https://www.ncbi.nlm.nih.gov/genome/?term=txid4232[orgn, and supplementary files.

## Abbreviations

A: Adenine
G: Guanine
C: Cytosine
U: Uracil
CAI: Codon adaptation index
ENC: Effective number of codons
GC: Total G + C contents of the gene
GC1, GC2, GC3: The G + C content at the first, second, third condon positions
GC12: The average GC content at the first and second condon positions
GC3s: The G + C content at the third position of synonymous codons
HaWRKY: *H. annuus* WRKY gene
NCBI: National Centre for Biotechnology
PR2: Parity Rule 2
RSCU: Relative synonymous codon usage
SCU: Synonymous codon usage
T3s, C3s, A3s, G3s: The content of T, C, A and G at the third condon position of synonymous codons
