# Supplementary material for: Analysis of codon usage bias of WRKY transcription factors in Helianthus annuus
Source: BMC Genom Data. 2022 Jun 20;23:46. doi: 10.1186/s12863-022-01064-8 (PMC9210703; doi:10.1186/s12863-022-01064-8)
Supplement: Supplementary file 1 — Additional file 1: Table S1. The composition indices values of codon usage in HaWRKY genome [file 12863_2022_1064_MOESM1_ESM.doc]

**Additional File 1**

**Table S1 The composition indices values of codon usage in HaWRKY genome**

| **Gene name** | **Gene ID** | **Protein accession NO.** | **T3s** | **C3s** | **A3s** | **G3s** | **GC3s** | **GC1** | **GC2** | **GC3** | **GC** | **CAI** | **ENC** |
| --- | --- | --- | --- | --- | --- | --- | --- | --- | --- | --- | --- | --- | --- |
| *HaWRKY1* | LOC110864423 | XP_021969172.1 | 0.4919 | 0.2642 | 0.3892 | 0.1862 | 0.333 | 0.4926 | 0.4458 | 0.3874 | 0.4419 | 0.223 | 52.57 |
| *HaWRKY2* | LOC110864505 | XP_021969276.1 | 0.4245 | 0.2406 | 0.4124 | 0.2500 | 0.366 | 0.4820 | 0.3921 | 0.3885 | 0.4209 | 0.213 | 52.89 |
| *HaWRKY3* | LOC110864606 | XP_021969411.1 | 0.3884 | 0.2277 | 0.4112 | 0.2713 | 0.378 | 0.4806 | 0.4240 | 0.4028 | 0.4358 | 0.213 | 56.95 |
| *HaWRKY4* | LOC110864810 | XP_021969654.1 | 0.3165 | 0.3669 | 0.4073 | 0.1632 | 0.427 | 0.4606 | 0.4956 | 0.4461 | 0.4674 | 0.224 | 50.15 |
| *HaWRKY5* | LOC110865300 | XP_021970237.1 | 0.3918 | 0.2577 | 0.4118 | 0.2222 | 0.371 | 0.3983 | 0.4689 | 0.3900 | 0.4191 | 0.190 | 50.43 |
| *HaWRKY6* | LOC110865842 | XP_021970864.1 | 0.4655 | 0.2069 | 0.3550 | 0.2210 | 0.337 | 0.5604 | 0.4628 | 0.3622 | 0.4618 | 0.218 | 52.55 |
| *HaWRKY7* | LOC110866620 | XP_021971454.1 | 0.3918 | 0.2653 | 0.4053 | 0.2269 | 0.377 | 0.4871 | 0.4000 | 0.3903 | 0.4258 | 0.218 | 52.81 |
| *HaWRKY8* | LOC110868000 | XP_021972771.1 | 0.3577 | 0.2269 | 0.4370 | 0.2423 | 0.367 | 0.4937 | 0.4686 | 0.3774 | 0.4465 | 0.183 | 57.02 |
| *HaWRKY9* | LOC110868001 | XP_021972772.1 | 0.3537 | 0.2683 | 0.4054 | 0.2512 | 0.402 | 0.4803 | 0.4375 | 0.4178 | 0.4452 | 0.190 | 53.70 |
| *HaWRKY10* | LOC110868376 | XP_021973216.1 | 0.4365 | 0.1694 | 0.4490 | 0.2122 | 0.294 | 0.4779 | 0.4468 | 0.3065 | 0.4104 | 0.197 | 50.80 |
| *HaWRKY11* | LOC110869273 | XP_021974263.1 | 0.4077 | 0.2333 | 0.4080 | 0.2583 | 0.370 | 0.4289 | 0.4248 | 0.3862 | 0.4133 | 0.196 | 53.35 |
| *HaWRKY12* | LOC110870911 | XP_021975670.1 | 0.4219 | 0.2148 | 0.3671 | 0.2643 | 0.371 | 0.5323 | 0.4369 | 0.3969 | 0.4554 | 0.191 | 52.40 |
| *HaWRKY13* | LOC110871776 | XP_021976202.1 | 0.5125 | 0.2125 | 0.2536 | 0.3333 | 0.400 | 0.4450 | 0.4000 | 0.4150 | 0.4200 | 0.228 | 53.63 |
| *HaWRKY14* | LOC110871877 | XP_021976320.1 | 0.3895 | 0.2105 | 0.3887 | 0.2647 | 0.373 | 0.5677 | 0.4688 | 0.3849 | 0.4738 | 0.197 | 54.24 |
| *HaWRKY15* | LOC110871889 | XP_021976334.1 | 0.3586 | 0.2590 | 0.3233 | 0.2955 | 0.441 | 0.4967 | 0.5199 | 0.4503 | 0.4890 | 0.223 | 55.90 |
| *HaWRKY16* | LOC110872305 | XP_021976767.1 | 0.4615 | 0.2186 | 0.3502 | 0.2598 | 0.360 | 0.4545 | 0.3896 | 0.3799 | 0.4080 | 0.213 | 49.01 |
| *HaWRKY17* | LOC110873218 | XP_021977846.1 | 0.3924 | 0.2691 | 0.3405 | 0.2690 | 0.416 | 0.4599 | 0.4545 | 0.4349 | 0.4498 | 0.201 | 58.70 |
| *HaWRKY18* | LOC110873546 | XP_021978177.1 | 0.4437 | 0.1953 | 0.3131 | 0.3060 | 0.390 | 0.5653 | 0.4656 | 0.4089 | 0.4800 | 0.209 | 51.96 |
| *HaWRKY19* | LOC110873608 | XP_021978260.1 | 0.4358 | 0.2793 | 0.4082 | 0.1736 | 0.352 | 0.4821 | 0.4420 | 0.3795 | 0.4345 | 0.210 | 51.25 |
| *HaWRKY20* | LOC110876056 | XP_021979929.1 | 0.4125 | 0.2417 | 0.4413 | 0.2183 | 0.344 | 0.4267 | 0.4104 | 0.3681 | 0.4017 | 0.183 | 54.00 |
| *HaWRKY21* | LOC110876057 | XP_021979930.1 | 0.4250 | 0.2167 | 0.4095 | 0.2732 | 0.358 | 0.4470 | 0.4139 | 0.3742 | 0.4117 | 0.176 | 53.09 |
| *HaWRKY22* | LOC110877484 | XP_021981327.1 | 0.3380 | 0.1667 | 0.4040 | 0.3102 | 0.381 | 0.4724 | 0.5276 | 0.3937 | 0.4646 | 0.200 | 48.76 |
| *HaWRKY23* | LOC110877496 | XP_021981338.1 | 0.3827 | 0.2654 | 0.3656 | 0.2548 | 0.402 | 0.5000 | 0.4429 | 0.4143 | 0.4524 | 0.224 | 59.15 |
| *HaWRKY24* | LOC110877782 | XP_021981679.1 | 0.4710 | 0.1935 | 0.4848 | 0.1901 | 0.279 | 0.4721 | 0.3807 | 0.2995 | 0.3841 | 0.193 | 55.71 |
| *HaWRKY25* | LOC110878107 | XP_021982058.1 | 0.4491 | 0.2037 | 0.4000 | 0.2586 | 0.340 | 0.4176 | 0.4176 | 0.3626 | 0.3993 | 0.183 | 57.68 |
| *HaWRKY26* | LOC110910181 | XP_021982085.1 | 0.4500 | 0.2136 | 0.4219 | 0.2346 | 0.331 | 0.4229 | 0.4122 | 0.3513 | 0.3955 | 0.183 | 55.00 |
| *HaWRKY27* | LOC110878727 | XP_021982775.1 | 0.3861 | 0.2405 | 0.5000 | 0.1739 | 0.320 | 0.4589 | 0.3913 | 0.3623 | 0.4042 | 0.178 | 49.39 |
| *HaWRKY28* | LOC110879102 | XP_021983199.1 | 0.4234 | 0.1870 | 0.3846 | 0.2869 | 0.363 | 0.5397 | 0.4175 | 0.3809 | 0.4460 | 0.234 | 51.20 |
| *HaWRKY29* | LOC110880030 | XP_021984272.1 | 0.4082 | 0.2449 | 0.3625 | 0.2573 | 0.390 | 0.4845 | 0.4255 | 0.4068 | 0.4389 | 0.212 | 51.49 |
| *HaWRKY30* | LOC110880954 | XP_021985060.1 | 0.4664 | 0.2108 | 0.4229 | 0.2111 | 0.310 | 0.3951 | 0.3776 | 0.3357 | 0.3695 | 0.210 | 49.93 |
| *HaWRKY31* | LOC110881115 | XP_021985172.1 | 0.4549 | 0.2196 | 0.4189 | 0.2201 | 0.328 | 0.4417 | 0.3834 | 0.3558 | 0.3937 | 0.189 | 53.81 |
| *HaWRKY32* | LOC110881332 | XP_021985312.1 | 0.5061 | 0.2065 | 0.4163 | 0.2040 | 0.298 | 0.3913 | 0.3602 | 0.3230 | 0.3582 | 0.232 | 49.36 |
| *HaWRKY33* | LOC110881463 | XP_021985403.1 | 0.3278 | 0.2665 | 0.4000 | 0.2443 | 0.406 | 0.4569 | 0.4831 | 0.4232 | 0.4544 | 0.205 | 56.54 |
| *HaWRKY34* | LOC110883348 | XP_021986826.1 | 0.3744 | 0.2603 | 0.4126 | 0.2323 | 0.381 | 0.4601 | 0.4348 | 0.3877 | 0.4275 | 0.214 | 61.00 |
| *HaWRKY35* | LOC110884041 | XP_021987406.1 | 0.4051 | 0.2372 | 0.3231 | 0.3198 | 0.425 | 0.4741 | 0.4138 | 0.4368 | 0.4416 | 0.188 | 51.56 |
| *HaWRKY36* | LOC110884300 | XP_021987700.1 | 0.4762 | 0.2585 | 0.4172 | 0.2028 | 0.335 | 0.4211 | 0.3732 | 0.3589 | 0.3844 | 0.256 | 45.85 |
| *HaWRKY37* | LOC110884380 | XP_021987783.1 | 0.4506 | 0.2332 | 0.3955 | 0.2277 | 0.343 | 0.4277 | 0.3899 | 0.3648 | 0.3941 | 0.209 | 51.68 |
| *HaWRKY38* | LOC110884382 | XP_021987784.1 | 0.4813 | 0.1992 | 0.4267 | 0.2059 | 0.298 | 0.4146 | 0.3639 | 0.3259 | 0.3681 | 0.215 | 46.89 |
| *HaWRKY39* | LOC110884429 | XP_021987831.1 | 0.4891 | 0.2432 | 0.4037 | 0.1987 | 0.324 | 0.3992 | 0.3782 | 0.3466 | 0.3746 | 0.238 | 49.04 |
| *HaWRKY40* | LOC110884449 | XP_021987852.1 | 0.3444 | 0.2365 | 0.2348 | 0.4131 | 0.514 | 0.4811 | 0.4948 | 0.5258 | 0.5006 | 0.179 | 50.52 |
| *HaWRKY41* | LOC110910436 | XP_021987853.1 | 0.4099 | 0.1943 | 0.3210 | 0.3543 | 0.417 | 0.4525 | 0.4274 | 0.4302 | 0.4367 | 0.169 | 51.64 |
| *HaWRKY42* | LOC110884726 | XP_021988131.1 | 0.4736 | 0.1923 | 0.4441 | 0.1837 | 0.284 | 0.4876 | 0.3981 | 0.3143 | 0.4000 | 0.187 | 48.33 |
| *HaWRKY43* | LOC110885886 | XP_021989298.1 | 0.4138 | 0.2759 | 0.2744 | 0.2658 | 0.435 | 0.4676 | 0.5185 | 0.4537 | 0.4799 | 0.214 | 55.47 |
| *HaWRKY44* | LOC110886005 | XP_021989449.1 | 0.4535 | 0.1922 | 0.3884 | 0.2351 | 0.333 | 0.5257 | 0.4486 | 0.3481 | 0.4408 | 0.222 | 53.31 |
| *HaWRKY45* | LOC110886021 | XP_021989466.1 | 0.3875 | 0.3000 | 0.3430 | 0.2277 | 0.415 | 0.4928 | 0.4353 | 0.4374 | 0.4552 | 0.216 | 56.78 |
| *HaWRKY46* | LOC110886183 | XP_021989639.1 | 0.3070 | 0.2412 | 0.3439 | 0.3255 | 0.459 | 0.4727 | 0.5018 | 0.4655 | 0.4800 | 0.214 | 50.61 |
| *HaWRKY47* | LOC110889630 | XP_021992883.1 | 0.4073 | 0.2419 | 0.2551 | 0.3305 | 0.455 | 0.5065 | 0.4967 | 0.4608 | 0.4880 | 0.232 | 51.04 |
| *HaWRKY48* | LOC110889731 | XP_021992989.1 | 0.4066 | 0.2527 | 0.4125 | 0.2368 | 0.369 | 0.4261 | 0.4261 | 0.3870 | 0.4130 | 0.190 | 50.49 |
| *HaWRKY49* | LOC110890231 | XP_021993517.1 | 0.4356 | 0.2466 | 0.4049 | 0.1548 | 0.322 | 0.5056 | 0.4340 | 0.3490 | 0.4295 | 0.202 | 51.68 |
| *HaWRKY50* | LOC110891153 | XP_021994511.1 | 0.3357 | 0.2679 | 0.4204 | 0.2655 | 0.407 | 0.4723 | 0.4461 | 0.4227 | 0.4470 | 0.193 | 56.29 |
| *HaWRKY51* | LOC110894011 | XP_021996867.1 | 0.3041 | 0.2488 | 0.2921 | 0.3711 | 0.502 | 0.4961 | 0.5116 | 0.5116 | 0.5065 | 0.220 | 49.24 |
| *HaWRKY52* | LOC110894274 | XP_021997170.1 | 0.4897 | 0.2069 | 0.3520 | 0.2810 | 0.358 | 0.4511 | 0.3913 | 0.3750 | 0.4058 | 0.240 | 53.01 |
| *HaWRKY53* | LOC110895238 | XP_021998208.1 | 0.3647 | 0.3059 | 0.3456 | 0.2286 | 0.425 | 0.4797 | 0.4606 | 0.4582 | 0.4662 | 0.204 | 58.27 |
| *HaWRKY54* | LOC110895693 | XP_021998708.1 | 0.3822 | 0.2471 | 0.3820 | 0.2267 | 0.380 | 0.5531 | 0.4823 | 0.3923 | 0.4759 | 0.225 | 55.76 |
| *HaWRKY55* | LOC110897944 | XP_022000372.1 | 0.5017 | 0.1847 | 0.3462 | 0.2490 | 0.330 | 0.5154 | 0.4034 | 0.3417 | 0.4202 | 0.229 | 51.67 |
| *HaWRKY56* | LOC110898958 | XP_022001505.1 | 0.4276 | 0.2780 | 0.3385 | 0.2162 | 0.387 | 0.5114 | 0.4545 | 0.4034 | 0.4564 | 0.218 | 56.49 |
| *HaWRKY57* | LOC110901781 | XP_022004259.1 | 0.4444 | 0.2275 | 0.4194 | 0.2167 | 0.336 | 0.4981 | 0.3385 | 0.3658 | 0.4008 | 0.203 | 47.13 |
| *HaWRKY58* | LOC110903575 | XP_022005112.1 | 0.4696 | 0.1887 | 0.3976 | 0.2281 | 0.315 | 0.5144 | 0.4179 | 0.3350 | 0.4224 | 0.224 | 52.77 |
| *HaWRKY59* | LOC110904963 | XP_022006517.1 | 0.4091 | 0.2727 | 0.3828 | 0.2228 | 0.378 | 0.4448 | 0.3880 | 0.3980 | 0.4103 | 0.208 | 50.37 |
| *HaWRKY60* | LOC110904964 | XP_022006518.1 | 0.4429 | 0.2557 | 0.3283 | 0.2663 | 0.393 | 0.4453 | 0.3869 | 0.4088 | 0.4136 | 0.230 | 49.63 |
| *HaWRKY61* | LOC110904966 | XP_022006519.1 | 0.4279 | 0.2488 | 0.3851 | 0.2547 | 0.373 | 0.4343 | 0.3825 | 0.3904 | 0.4024 | 0.209 | 49.49 |
| *HaWRKY62* | LOC110904967 | XP_022006520.1 | 0.4352 | 0.2228 | 0.3728 | 0.2830 | 0.374 | 0.4426 | 0.3934 | 0.3975 | 0.4112 | 0.227 | 48.96 |
| *HaWRKY63* | LOC110906043 | XP_022007075.1 | 0.4351 | 0.2678 | 0.3188 | 0.2332 | 0.391 | 0.4479 | 0.4792 | 0.4062 | 0.4444 | 0.236 | 56.33 |
| *HaWRKY64* | LOC110907852 | XP_022008465.1 | 0.4515 | 0.2025 | 0.3881 | 0.2304 | 0.332 | 0.5190 | 0.3979 | 0.3564 | 0.4245 | 0.184 | 50.79 |
| *HaWRKY65* | LOC110907968 | XP_022008574.1 | 0.4457 | 0.2434 | 0.4059 | 0.2061 | 0.341 | 0.4911 | 0.4112 | 0.3580 | 0.4201 | 0.225 | 53.07 |
| *HaWRKY66* | LOC110910002 | XP_022010411.1 | 0.4298 | 0.2355 | 0.3964 | 0.2537 | 0.362 | 0.3943 | 0.3975 | 0.3912 | 0.3943 | 0.191 | 54.73 |
| *HaWRKY67* | LOC110910044 | XP_022010446.1 | 0.4218 | 0.2857 | 0.4610 | 0.1973 | 0.348 | 0.4245 | 0.3491 | 0.3679 | 0.3805 | 0.217 | 55.16 |
| *HaWRKY68* | LOC110911061 | XP_022011318.1 | 0.3633 | 0.2837 | 0.3778 | 0.2706 | 0.422 | 0.5435 | 0.3832 | 0.4348 | 0.4538 | 0.221 | 61.00 |
| *HaWRKY69* | LOC110911702 | XP_022012026.1 | 0.4664 | 0.2569 | 0.3640 | 0.1955 | 0.350 | 0.4923 | 0.4365 | 0.3746 | 0.4345 | 0.227 | 52.49 |
| *HaWRKY70* | LOC110911726 | XP_022012045.1 | 0.3333 | 0.3120 | 0.3543 | 0.2995 | 0.468 | 0.5227 | 0.4708 | 0.4870 | 0.4935 | 0.227 | 58.21 |
| *HaWRKY71* | LOC110912297 | XP_022012682.1 | 0.3929 | 0.1965 | 0.3447 | 0.3361 | 0.412 | 0.5635 | 0.4385 | 0.4286 | 0.4769 | 0.208 | 52.89 |
| *HaWRKY72* | LOC110915202 | XP_022015554.1 | 0.2615 | 0.2661 | 0.3850 | 0.3430 | 0.473 | 0.4305 | 0.4007 | 0.4901 | 0.4404 | 0.141 | 54.77 |
| *HaWRKY73* | LOC110915420 | XP_022015811.1 | 0.4456 | 0.1531 | 0.2820 | 0.3560 | 0.394 | 0.5014 | 0.4672 | 0.4103 | 0.4596 | 0.231 | 48.57 |
| *HaWRKY74* | LOC110915480 | XP_022015882.1 | 0.3925 | 0.2491 | 0.3164 | 0.2780 | 0.418 | 0.5185 | 0.4722 | 0.4259 | 0.4722 | 0.191 | 55.20 |
| *HaWRKY75* | LOC110915516 | XP_022015923.1 | 0.4615 | 0.2333 | 0.3983 | 0.1888 | 0.326 | 0.4438 | 0.4601 | 0.3436 | 0.4158 | 0.230 | 49.38 |
| *HaWRKY76* | LOC110915928 | XP_022016370.1 | 0.3514 | 0.3213 | 0.3287 | 0.2287 | 0.446 | 0.5076 | 0.4788 | 0.4636 | 0.4834 | 0.226 | 54.84 |
| *HaWRKY77* | LOC110917147 | XP_022017441.1 | 0.3473 | 0.3588 | 0.3509 | 0.2074 | 0.448 | 0.4560 | 0.4843 | 0.4591 | 0.4665 | 0.255 | 51.42 |
| *HaWRKY78* | LOC110917201 | XP_022017486.1 | 0.4574 | 0.2092 | 0.4492 | 0.1667 | 0.289 | 0.4561 | 0.4198 | 0.3206 | 0.3989 | 0.187 | 49.54 |
| *HaWRKY79* | LOC110917334 | XP_022017600.1 | 0.3632 | 0.2691 | 0.4256 | 0.2320 | 0.383 | 0.4599 | 0.4051 | 0.3978 | 0.4209 | 0.189 | 57.06 |
| *HaWRKY80* | LOC110919788 | XP_022019736.1 | 0.3392 | 0.2731 | 0.4198 | 0.2525 | 0.403 | 0.4844 | 0.4533 | 0.4221 | 0.4533 | 0.193 | 56.51 |
| *HaWRKY81* | LOC110921717 | XP_022019736.1 | 0.4915 | 0.2288 | 0.3578 | 0.1739 | 0.317 | 0.5034 | 0.4384 | 0.3322 | 0.4247 | 0.245 | 51.17 |
| *HaWRKY82* | LOC110922561 | XP_022022538.1 | 0.4845 | 0.2519 | 0.4043 | 0.1822 | 0.323 | 0.4760 | 0.4012 | 0.3443 | 0.4072 | 0.252 | 51.16 |
| *HaWRKY83* | LOC110922702 | XP_022022625.1 | 0.3717 | 0.2906 | 0.4261 | 0.1716 | 0.367 | 0.4328 | 0.4517 | 0.3845 | 0.4230 | 0.236 | 48.91 |
| *HaWRKY84* | LOC110922861 | XP_022022754.1 | 0.4192 | 0.2020 | 0.4599 | 0.2458 | 0.332 | 0.4867 | 0.3384 | 0.3536 | 0.3929 | 0.190 | 46.55 |
| *HaWRKY85* | LOC110922953 | XP_022022840.1 | 0.4201 | 0.2365 | 0.3551 | 0.2734 | 0.390 | 0.5123 | 0.4444 | 0.4444 | 0.4671 | 0.216 | 56.11 |
| *HaWRKY86* | LOC110926168 | XP_022025614.1 | 0.4486 | 0.1981 | 0.3533 | 0.2894 | 0.371 | 0.5691 | 0.4353 | 0.3941 | 0.4662 | 0.202 | 56.58 |
| *HaWRKY87* | LOC110928824 | XP_022027560.1 | 0.3556 | 0.2675 | 0.4286 | 0.2310 | 0.383 | 0.5059 | 0.4299 | 0.3943 | 0.4434 | 0.240 | 58.07 |
| *HaWRKY88* | LOC110929319 | XP_022028154.1 | 0.5282 | 0.1831 | 0.4322 | 0.1909 | 0.272 | 0.4804 | 0.3743 | 0.2905 | 0.3818 | 0.228 | 52.49 |
| *HaWRKY89* | LOC110929731 | XP_022028611.1 | 0.4556 | 0.2620 | 0.3683 | 0.1777 | 0.346 | 0.5333 | 0.4500 | 0.3630 | 0.4488 | 0.224 | 53.02 |
| *HaWRKY90* | LOC110930168 | XP_022029096.1 | 0.4444 | 0.2698 | 0.4036 | 0.2278 | 0.366 | 0.4553 | 0.3211 | 0.3821 | 0.3862 | 0.222 | 53.10 |
| *HaWRKY91* | LOC110930318 | XP_022029302.1 | 0.3404 | 0.2596 | 0.3223 | 0.2836 | 0.444 | 0.4522 | 0.5478 | 0.4522 | 0.4841 | 0.235 | 49.01 |
| *HaWRKY92* | LOC110930372 | XP_022029366.1 | 0.2907 | 0.3178 | 0.4206 | 0.2765 | 0.451 | 0.4632 | 0.4294 | 0.4663 | 0.4530 | 0.199 | 51.22 |
| *HaWRKY93* | LOC110930373 | XP_022029367.1 | 0.3480 | 0.2800 | 0.4648 | 0.2132 | 0.376 | 0.4369 | 0.4239 | 0.3948 | 0.4186 | 0.210 | 49.84 |
| *HaWRKY94* | LOC110930672 | XP_022029716.1 | 0.3636 | 0.2998 | 0.3644 | 0.2225 | 0.414 | 0.4832 | 0.4458 | 0.4359 | 0.4550 | 0.214 | 53.89 |
| *HaWRKY95* | LOC110936369 | XP_022034430.1 | 0.4744 | 0.1907 | 0.3923 | 0.2601 | 0.332 | 0.4559 | 0.3934 | 0.3603 | 0.4032 | 0.230 | 47.28 |
| *HaWRKY96* | LOC110936976 | XP_022035075.1 | 0.4928 | 0.1993 | 0.3115 | 0.2612 | 0.354 | 0.4496 | 0.4236 | 0.3718 | 0.4150 | 0.200 | 47.24 |
| *HaWRKY97* | LOC110937195 | XP_022035280.1 | 0.4484 | 0.2645 | 0.3521 | 0.2305 | 0.371 | 0.4794 | 0.4433 | 0.3840 | 0.4356 | 0.237 | 54.73 |
| *HaWRKY98* | LOC110937240 | XP_022035328.1 | 0.3509 | 0.2632 | 0.3945 | 0.2670 | 0.409 | 0.4966 | 0.4796 | 0.4320 | 0.4694 | 0.188 | 55.29 |
| *HaWRKY99* | LOC110937455 | XP_022035569.1 | 0.4167 | 0.2000 | 0.4549 | 0.2030 | 0.310 | 0.4987 | 0.4465 | 0.3290 | 0.4247 | 0.189 | 45.82 |
| *HaWRKY100* | LOC110937474 | XP_022035591.1 | 0.4563 | 0.2460 | 0.3958 | 0.1931 | 0.338 | 0.4970 | 0.4329 | 0.3567 | 0.4289 | 0.213 | 47.21 |
| *HaWRKY101* | LOC110937590 | XP_022035726.1 | 0.3285 | 0.3577 | 0.3699 | 0.1949 | 0.443 | 0.4659 | 0.4837 | 0.4599 | 0.4698 | 0.243 | 49.99 |
| *HaWRKY102* | LOC110938156 | XP_022036318.1 | 0.3438 | 0.3099 | 0.3261 | 0.2557 | 0.454 | 0.5060 | 0.4759 | 0.4719 | 0.4846 | 0.209 | 55.03 |
| *HaWRKY103* | LOC110939562 | XP_022036810.1 | 0.4436 | 0.2105 | 0.3525 | 0.2931 | 0.378 | 0.4529 | 0.4000 | 0.3941 | 0.4157 | 0.233 | 48.37 |
| *HaWRKY104* | LOC110939612 | XP_022036883.1 | 0.3279 | 0.2737 | 0.3725 | 0.2814 | 0.437 | 0.5180 | 0.4352 | 0.4671 | 0.4735 | 0.252 | 57.32 |
| *HaWRKY105* | LOC110940633 | XP_022037876.1 | 0.2900 | 0.2684 | 0.3155 | 0.3385 | 0.492 | 0.4697 | 0.5492 | 0.4962 | 0.5051 | 0.216 | 54.65 |
| *HaWRKY106* | LOC110941958 | XP_022039347.1 | 0.3286 | 0.2911 | 0.3920 | 0.2414 | 0.420 | 0.4504 | 0.4743 | 0.4338 | 0.4528 | 0.202 | 53.94 |
| *HaWRKY107* | LOC110942188 | XP_022039626.1 | 0.4177 | 0.2595 | 0.3356 | 0.2380 | 0.392 | 0.5407 | 0.4525 | 0.4153 | 0.4695 | 0.223 | 54.04 |
| *HaWRKY108* | LOC110942625 | XP_022040092.1 | 0.3807 | 0.2437 | 0.3657 | 0.2619 | 0.398 | 0.5144 | 0.4362 | 0.4280 | 0.4595 | 0.224 | 53.39 |
| *HaWRKY109* | LOC110945138 | XP_022042456.1 | 0.4977 | 0.1689 | 0.3399 | 0.2670 | 0.331 | 0.4348 | 0.4239 | 0.3514 | 0.4034 | 0.162 | 43.47 |
| *HaWRKY110* | LOC110910181 | XP_022010582 | 0.4500 | 0.2136 | 0.4219 | 0.2346 | 0.331 | 0.4229 | 0.4122 | 0.3513 | 0.3955 | 0.183 | 55.00 |
| *HaWRKY111* | LOC110945166 | XP_022042463 | 0.5000 | 0.2108 | 0.3050 | 0.2815 | 0.367 | 0.4272 | 0.3568 | 0.4038 | 0.3959 | 0.193 | 50.61 |
| *HaWRKY112* | LOC118485943 | XP_035838379 | 0.3957 | 0.2727 | 0.3801 | 0.2298 | 0.388 | 0.4580 | 0.4832 | 0.4160 | 0.4524 | 0.217 | 55.01 |
| *HaWRKY113* | LOC110885084 | XP_021988476.1 | 0.4561 | 0.1957 | 0.3888 | 0.2485 | 0.333 | 0.4584 | 0.3730 | 0.3498 | 0.3937 | 0.183 | 52.17 |
| *HaWRKY114* | LOC118488941 | XP_035842278 | 0.4167 | 0.2056 | 0.3681 | 0.3018 | 0.383 | 0.4534 | 0.3941 | 0.3983 | 0.4153 | 0.198 | 53.38 |
| *HaWRKY115* | LOC110917058 | XP_022017371 | 0.3969 | 0.1938 | 0.3353 | 0.3780 | 0.435 | 0.5341 | 0.3955 | 0.4477 | 0.4591 | 0.187 | 55.14 |
| Mean value |  |  | 0.4124 | 0.2420 | 0.3794 | 0.2481 | 0.376 | 0.4755 | 0.4311 | 0.3960 | 0.4342 | 0.210 | 52.63 |
